# *Babesia* and *Theileria* Identification in Adult Ixodid Ticks from Tapada Nature Reserve, Portugal

**DOI:** 10.3390/pathogens11020222

**Published:** 2022-02-08

**Authors:** Nélida Fernández, Belen Revuelta, Irene Aguilar, Jorge Francisco Soares, Annetta Zintl, Jeremy Gray, Estrella Montero, Luis Miguel Gonzalez

**Affiliations:** 1 Facultad de Veterinaria, Alfonso X el Sabio University, 28691 Madrid, Spain; nfernpat@uax.es (N.F.); i.aguilar.garcia@hotmail.com (I.A.); 2 Parasitology Reference and Research Laboratory, Centro Nacional de Microbiología, Instituto de Salud Carlos III, Majadahonda, 28220 Madrid, Spain; belen.revuelta@isciii.es (B.R.); lmgonzal@isciii.es (L.M.G.); 3 Wild Animal Health, BeWild Conservation Medicine, 1600-646 Lisboa, Portugal; conservationmedicine@be-wild.org; 4 UCD School of Veterinary Sciences, University College Dublin, D04 W6F6 Dublin, Ireland; annetta.zintl@ucd.ie; 5 UCD School of Biology and Environmental Science, University College Dublin, D04 N2E5 Dublin, Ireland; jeremy.gray@ucd.ie

**Keywords:** *Babesia*, host blood analysis, fallow deer, ixodid ticks, piroplasm, red deer, *Theileria*

## Abstract

This study, conducted in a nature reserve in southern Portugal, investigated the frequency and diversity of tick-borne piroplasms in six species of adult ixodid ticks removed from 71 fallow deer (*Dama dama*) and 12 red deer (*Cervus elaphus*), collected over the period 2012–2019. The majority of 520 ticks were *Ixodes ricinus* (78.5%), followed by *Rhipicephalus sanguineus* sensu lato, *Hyalomma lusitanicum, Haemaphysalis punctata, Dermacentor marginatus*, and *Ixodes hexagonus.* The *R. sanguineus* ticks collected from the deer were clearly exophilic, in contrast to the endophilic species usually associated with dogs. Four tick-borne piroplasms, including *Theileria* spp., and the zoonotic species, *Babesia divergens* and *Babesia microti,* were detected. *B. divergens* 18S rDNA, identical to that of the bovine reference strain U16370 and to certain strains from red deer, was detected in *I. ricinus* ticks removed from fallow deer. The sporadic detection of infections in ticks removed from the same individual hosts suggests that the piroplasms were present in the ticks rather than the hosts. *Theileria* sp. OT3 was found in *I. ricinus* and, along with *T. capreoli,* was also detected in some of the other tick species. The natural vector and pathogenic significance of this piroplasm are unknown.

## 1. Introduction

The most common tick-borne diseases (TBDs) of humans in Europe (Lyme borreliosis and tick-borne encephalitis) are notifiable to the European Centre for Disease Prevention and Control (ECDC). However, diseases caused by less common tick-borne pathogens (TBPs), such as *Babesia*, *Theileria*, and *Rickettsia* species, although representing potential health risks for humans, domestic animals, or wildlife, are not included in current surveillance schemes. In the absence of relevant surveillance schemes, molecular analysis of ticks collected in areas where humans and animals are exposed to tick bites can detect endemic TBPs; identify new ones; and, alongside reported human cases and serosurveys, contribute to epidemiological information.

The climate, flora, and fauna of Tapada Nacional de Mafra, where the present study was carried out, constitute a potential environment for ixodid ticks and TBPs. From 2012 to 2019, several species of adult ticks were collected from parasitized fallow (*Dama dama*) and red deer (*Cervus elaphus)* during the spring and autumn seasons, when most tick host-seeking activity occurs. The tick species involved can transmit and maintain several viral, bacterial, and protozoan pathogens of public health and veterinary importance [1,2,3,4,5,6]. In this study, we focused on the detection of piroplasm protozoans of the *Babesia* and *Theileria* genera. These parasites infect erythrocytes (and also lymphocytes in the case of *Theileria* spp.) of a variety of vertebrate hosts, but particularly cervids, which are prominent components of the Tapada Nature Reserve fauna.

Of particular interest were the zoonotic species *Babesia divergens* and *B. venatorum,* which belong to the *Babesia* sensu stricto (s.s.) group (Clade X [7]), and are transmitted by *Ixodes ricinus,* the castor bean tick, the most widespread ixodid species in Europe [8]. While roe deer (*Capreolus capreolus*) are considered the natural hosts of *B. venatorum* [9,10], cattle are the main reservoirs of *B. divergens* and frequently suffer clinical disease (redwater fever) as a consequence of infection [11]. *B. divergens*-like DNA sequences have also been reported in red deer, roe deer, reindeer (*Rangifer tarandus*), and fallow deer [9,12,13,14,15,16,17,18], but there is some uncertainty about the precise identity of these parasites and, so far, there is no evidence that *Babesia* isolates from deer are infectious for either humans or cattle [12,19].

*Babesia microti,* which is also potentially zoonotic, particularly in the USA [20], is distinct from the *Babesia* s.s. group and consists of a species complex divided into five distinct clades, infecting a wide range of vertebrates with the notable exception of ungulates [21]. Those in clade 1, also referred to as *B. microti* sensu stricto (s.s.), cause most of the human babesiosis cases worldwide, and those in clade 3 include the *B. microti* Munich strain, which has an ambiguous zoonotic status [21]. In Europe, both *B. microti* s.s. strains and the *B. microti* Munich strain have been detected in *I. ricinus* and in several mammal species, particularly rodents. On the other hand, few cases of human babesiosis have been reported in Europe so far [22], which appears to be at odds with the frequent occurrence of the parasites in small mammals.

*Theileria* species are known to be transmitted by ixodid ticks of the genera *Amblyomma, Haemaphysalis*, *Hyalomma*, and *Rhipicephalus* [23], and the recent detection of DNA of *Theileria* spp., such as *Theileria* OT3 in *I. ricinus*, suggests that this tick species might also be a *Theileria* vector [4,24]. *Theileria* spp. are not considered zoonotic [25], but some have a major impact on the livestock industry, especially in tropical and sub-tropical countries [26], while others can infect wild animals including red deer [27,28].

This study, focused on a sample of 520 adult ticks collected from fallow and red deer in Tapada Nature Reserve, was conducted to determine the diversity and relative abundance of the tick fauna, and their possible roles in the maintenance and transmission of *Babesia* and *Theileria* parasites by the detection and amplification of the 18S rRNA and cytochrome c oxidase subunit I (COI) genes.

## 2. Results

### 2.1. Ticks Removed from Deer

**Figure 1:**
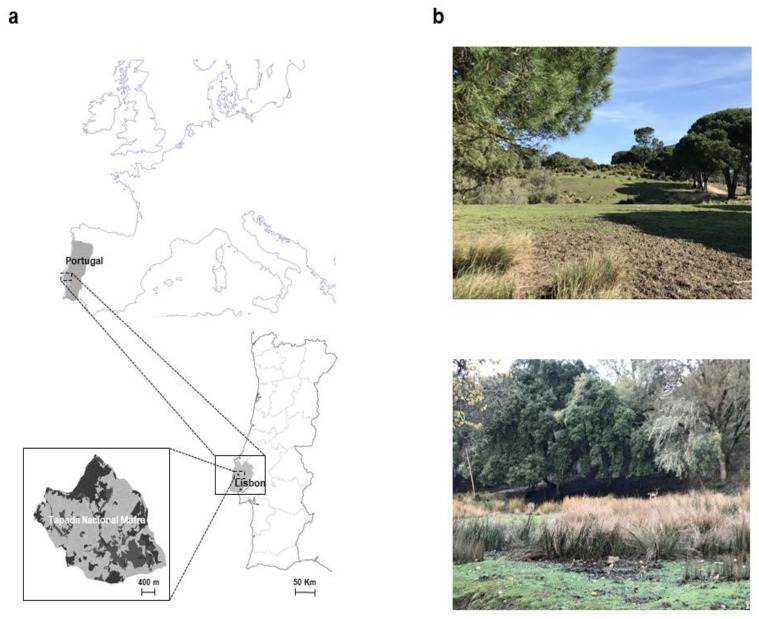
The locations of Tapada Nacional de Mafra and examples of sample sites. (**a**) The location of Tapada Nacional de Mafra in Europe. (**b**) Pictures show different areas of Tapada Nacional de Mafra inhabited by fallow and red deer.

During the period 2012–2019, a total of 520 adult ticks were collected from May to September in Tapada Nacional de Mafra, Portugal (Figure 1).

Of this total, 350 engorged female (67.3%) and 114 male ticks (21.9%) were removed from 71 fallow deer, and 27 engorged female (5.19%) and 29 (5.58%) male ticks from 12 red deer. Most of the identified ticks removed from fallow deer were *I. ricinus* (n = 377, 81.2%, *p* < 0.05), followed by *Rhipicephalus sanguineus* sensu lato (s.l.) (n = 42, 9.0%), *Hyalomma lusitanicum* (n = 22, 4.7%), *Haemaphysalis punctata* (n = 15, 3.2%), *Dermacentor marginatus* (n = 6, 1.3%), and *Ixodes hexagonus* (n = 2, 0.4%). Ticks removed from red deer were identified as *I. ricinus* (n = 31, 55.4%), again the most abundant tick (*p* < 0.05), followed by *R. sanguineus* s.l., (n = 23, 41.1%) and *D. marginatus* (n = 2, 3.6%) (Table 1).

### 2.2. Piroplasm Infection and Coinfection

Out of the 520 ticks tested, only 75 specimens of *I. ricinus*, *R. sanguineus* s.l., and *H. lusitanicum* were found to be infected with piroplasms. *B. divergens, T. capreoli*, and *Theileria* sp. OT3 were detected by real-time polymerase chain reaction (PCR) and sequencing of part of the 18S rRNA gene (408–430 bp) [30,31], with *Theileria* sp. OT3 being the most abundant (*p* < 0.05). In addition, two different *B. microti* 18S rDNA fragments of 157 and 155 bp were detected by using a highly sensitive real-time PCR designed for the diagnosis of *B. microti* human babesiosis [32].

In particular, of the 377 *I. ricinus* samples removed from fallow deer, 8 (2.1%) were positive for *B. divergens* and 25 (6.6%) for *B. microti*. The *B. microti* 18S rDNA fragment of 157 bp was amplified in 7 ticks, while the fragment of 155 bp was amplified in another 18 ticks. *I. ricinus* was also infected with *T. capreoli* (n = 10, 2.7%) and *Theileria* sp. OT3 (n = 31, 8.3%) (Table 2).

Co-infections involving *B. microti* with the following species were as follows: 

*B. divergens* (n = 2), *T. capreoli* (n = 2), *Theileria* sp. OT3 (n = 6), and *T. capreoli* /*Theileria* sp. OT3 (n = 1). Coinfections with *B. divergens*/*Theileria* sp.OT3 (n = 1) and *T. capreoli*/*Theileria* sp. OT3 (n = 1) were also detected (Table 2).

Of the 31 *I. ricinus* samples collected from red deer, 5 (16.1%) tested positive for *T. capreoli* (Table 2). Of the 42 *R. sanguineus* s.l. ticks collected from fallow deer, 3 (7.1%) were positive for *B. microti* (155 bp fragment) and 2 (4.8%) for *Theileria* sp. OT3. One *B. microti*/*Theileria* sp. OT3 coinfection was also detected. Of the 23 *R. sanguineus* s.l. ticks collected from red deer, 4.3% were positive for *T. capreoli* (n = 1) and *Theileria* sp. OT3 (n = 1), respectively.

Finally, of the 22 *H. lusitanicum* samples removed from fallow deer, 2 (9.1%) were found to be infected with *B. microti* (155 bp fragment). We did not detect co-infections in ticks collected from red deer or infected *H. lusitanicum* (Table 2).

Of note, in most cases (63%), only one of several ticks removed from the same deer tested positive for any one of the piroplasms (Supplementary Tables S1 and S2).

#### 2.2.1. *Babesia* Spp. Typing

**Figure 2:**
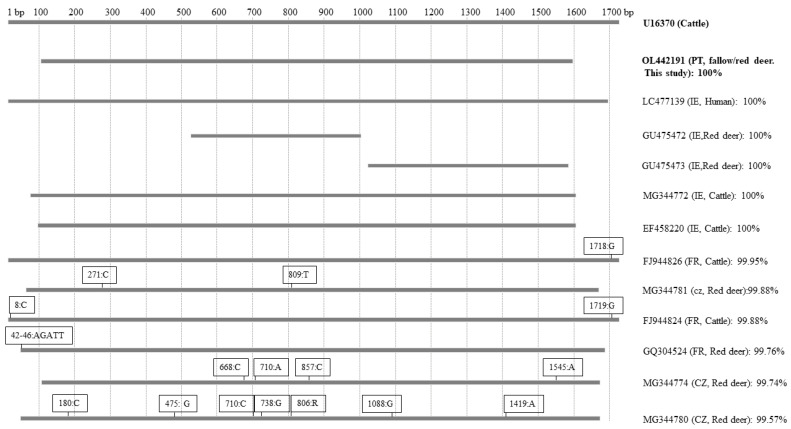
The relative length, positions, and heterologies of 18S rDNA gene sequences of *Babesia* *divergens* obtained in this work, together with others from cattle, human, and red deer compared to the reference sequence U16370. Of the 13 *B. divergens* isolates used to compare regions of the 18S rRNA gene, the sequences obtained in this work (QL442191) were 100% identical to that of the reference bovine isolate (U16370) and to those of several others from cattle and red deer, and also to one human isolate. However, most other published red deer sequences differed from QL442191 and the bovine *B. divergens* reference strain by up to 7 nucleotides in various positions. The numbers and letters refer to positions and nucleotide bases that are different from the reference sequence. Identity scores are made according to Clustal Omega.

An almost entire sequence (1492 bp) of the *B. divergens* 18S rRNA gene was obtained from four of the eight infected *I. ricinus* ticks that were removed from fallow deer. No nucleotide variations were detected among these sequences, which were 100% identical to several entire human and bovine isolates including the bovine isolate GenBank: U16370, widely used as a reference [33] (Figure 2). The presence of *B. capreoli,* a very closely related species to *B. divergens* commonly associated with deer [12], was ruled out by both the 18S rDNA analysis [19] and also by amplification of part (234 bp) of the COI gene, which confirmed the identity of the piroplasms as *B. divergens*.

The *B. microti* 157 bp fragment was 100% identical to the *B. microti* 18S rRNA gene of seven different sequences in GenBank, five of which had been identified as the *B. microti* Munich strain. The 155 bp fragment was 100% identical to the *B. microti* 18S rDNA from a number of human and *Ixodes* tick isolates of *B. microti* (n = 47), including the *B. microti* isolate Jena EF413181 responsible for the autochthonous human *B. microti* infection that occurred in Germany [34] (Supplementary Figure S1). To provide phylogenetic analysis of the two *B. microti* isolates, PCR reactions for amplifying larger fragments of the 18S rRNA gene and also part of the COI gene were performed [35], but did not yield any products. The limitation in obtaining large fragments of the gene could be associated with a low number of parasites present in ticks and an inverse correlation between the efficiency of the amplification and the size of the fragment.

#### 2.2.2. *Theileria* Spp. Typing

To obtain more sequence information about the *Theileria* isolates, we amplified and sequenced almost the entire 18S rRNA gene. The 18S rDNA sequence (1230 bp length) obtained from 1 *I. ricinus* of 16 ixodid ticks infected with *T. capreoli* was 100% identical to 3 *T. capreoli* 18S rDNA isolates in the databank, including one associated with the first theileriosis case detected in Spain in a red deer imported from Northern Europe (GenBank: AY421708.1) [36].

The 18S rDNA sequences obtained from 4 *I. ricinus* ticks out of 33 infected with *Theileria* sp. OT3 (1218 bp) were 100% identical to each other and to two *Theileri* sp. 18S rDNA records in GenBank, while one of them was associated with the first report on the occurrence of *Theileria* sp. OT3 in sheep in China (KF470868) [37]. These nucleotide sequences from Tapada and China differed by one or two nucleotides (99.92–99.84%) from *Theileria* sp. OT3 18S rDNA sequences from sheep, chamois, red deer, and roe deer from Spain [28].

## 3. Discussion

Six different tick species were collected from fallow and red deer in Tapada, Portugal. These tick species occur across the Iberian Peninsula [15,29,38,39,40,41] and five of them, *I. ricinus*, *R. sanguineus*, *H. lusitanicum*, *H. punctata*, and *D. marginatus*, have been previously associated with fallow and red deer in Portugal [29]. The finding of the hedgehog tick, *I. hexagonus,* on fallow deer is unusual, though it has been reported previously on roe deer [42].

Some of the tick species collected (e.g., *R. sanguineus* s.l.*, D. marginatus*, and *H. lusitanicum*) are regarded as adapted to drier habitats than occur in the reserve. It was, therefore, surprising to find them in the wet habitat and fauna of Tapada where they coexist with *I. ricinus,* which, as the most common species in Atlantic climatic regions [38], was the most abundant, as expected.

*B. divergens* was detected in 2.1% of *I. ricinus* specimens, which is similar to infection rates for adult ticks elsewhere in Europe [43]. The pathogen’s identity was determined by detection of the almost complete 18S rRNA gene, which was identical to the bovine isolate U16370 and differed slightly from the closely related cervine species, *B. capreoli*, [19]. Specific amplification of part of the *B. divergens* cytochrome c oxidase subunit I (COI) gene unequivocally supported the 18S rRNA gene differentiation of these *B. divergens* sequences from *B. capreoli.* The apparent absence of *B. capreoli* is perhaps surprising considering that it is regarded as a cervid *Babesia* species. However, it should be noted that roe deer, possibly its primary host, are not present in this habitat, which also explains the absence of *Babesia venatorum.*

It is curious that the *B. divergens* sequences were only detected in ticks removed from fallow deer, considering that the only previous reports of complete identity of *B. divergens* in deer with U16370 have been in occasional samples from red rather than fallow deer (e.g., [16] and GenBank accession numbers MH697659, KX018019, MT151377, MN563158, and GQ304524). This apparent association of *B. divergens*-infected ticks with fallow deer is difficult to interpret, considering that red deer were also present in the habitat, but did not yield *B. divergens*-infected ticks, though it should be noted that the number of *I. ricinus* obtained from fallow deer was far higher than from red deer (371 versus 31). *B. divergens*-like parasites have rarely been reported from fallow deer, as reports either provide no sequence data [15,44] or only partial sequences [GenBank Accession Numbers KY242395, KY242396] which show low homology with the bovine reference strain U16370. It, therefore, appears probable that the detected pathogen was present in the ticks rather than in the fallow deer on which they were feeding. Since there are no cattle near the study site, red deer remain the most plausible source of these *B. divergens* sequences, which showed 100% identity to two long 18S rDNA sequences of 1639 bp and 1648 bp deposited in GenBank (GQ304524, GQ304525) obtained from red deer spleen. It is notable that the infected ticks were collected from deer that were mostly a source of uninfected ticks, again suggesting that the infections were present in the ticks rather than the deer, having been acquired by the previous tick generation and persisted in the collected ticks.

It is important to point out that there is, in fact, still no unequivocal evidence that *B. divergens*-like piroplasms from deer can cause human babesiosis or, indeed, even infect cattle, and it is interesting that, in a recent study, *B. divergens* was not detected in cattle that were in close proximity to infected deer [45]. The 18S rRNA gene is probably not the optimal choice for discrimination of *B. divergens* genotypes, although sequences of this gene are the most widely available [22]. Cross transmission studies are required to further explore the host specificity of *B. divergens* from deer, including the use of gerbils *(Meriones unguiculatus)* as proxies for humans [46]. So far, transmission experiments have not resulted in established infections, except in deer that had been splenectomized [11].

The other *Babesia* species identified in this study was *B. microti,* DNA fragments of which were detected in *I. ricinus, R. sanguineus* s.l., and *H. lusitanicum* adult ticks. While *I. ricinus* is a recognized vector of *B. microti* [47], *R. sanguineus* s.l. does not transmit the parasite [48]. So far, the uncommon *H. lusitanicum*–*B. microti* association, although previously detected in Spain, has not been supported by vector competence evidence either [15]. Based on the 18S rRNA gene fragments obtained in this study, two different *B. microti* strains may infect *I. ricinus* ticks in Tapada. One strain is evidently related to clade 1, to which zoonotic North American genotypes belong, and the second to the Munich strain, which belongs to clade 3 [21]. This latter genotype was once thought to be transmitted only by *I. trianguliceps*, which does not bite humans, but it has been detected in the anthropophilic *I. ricinus* [49,50,51] and has been associated with asymptomatic and moderate human babesiosis in Europe [52,53]. It seems, therefore, that zoonotic forms of *B. microti* might occur in Tapada, and to investigate this further, we are currently validating methods, such as the MinION long-read sequencing technology, to generate full-length nucleotide sequences of different molecular markers.

The detection in *I. ricinus* of DNA of *Theileria* sp OT3 and *T. capreoli,* associated with sheep and red deer, respectively, in China and Spain, supports previous suggestions that this tick species is a vector of certain *Theileria* spp. [14,24,54,55,56]. No sheep are observed in the immediate vicinity of Tapada and it is probable that deer are the reservoir hosts for the *Theileria* species detected in this study.

The co-existence of different piroplasms within the same tick in Tapada added an additional layer of complexity to the analysis of the tick–host relationships and pathogen transmission. Coinfections could be due to blood feeding on different vertebrate hosts or through co-feeding [3,57], although the latter is less likely to consider the small number of deer that carried several infected ticks at the same time. However, this hypothesis demands a more comprehensive evaluation and robust co-infection models [3,58].

## 4. Conclusions

This study shows the presence of *Babesia* and *Theileria* DNA in ticks from Tapada and suggests certain associations between infected ticks and susceptible hosts. The finding of *B. divergens* DNA identical to sequences from cattle supports observations made previously for a small proportion of sequences from red deer, and has possible implications for the role of these hosts in bovine and human babesiosis. However, at present, there are no transmission or epidemiological studies which suggest that the *B. divergens* strains detected in deer are infective for humans or cattle. Another interesting outcome of the study is the possible association of *Theileria* spp. with *I. ricinus,* which has not been firmly established as a vector of these piroplasms. The detection of *B. microti* in the ticks is not surprising, since this species complex is very widespread in Europe as a parasite of small mammals, but the fragments of *B. microti* DNA obtained were too small to draw any firm conclusions about the presence of zoonotic strains of this parasite in the reserve. Future studies should focus on alternatives to the 18S rRNA gene for pathogen identification, and should be combined with innovative sensitive and specific blood meal analysis methods, such as the use of retrotransposons in qPCR assays [59] in both fed and unfed ticks, to obtain a more complete picture of tick-borne pathogen epidemiology in complex habitats such as Tapada Nature Reserve.

## 5. Materials and Methods

### 5.1. Tick Samples

A total of 520 adult ticks were manually removed from 71 fallow deer (*D. dama*) and 12 red deer (*C. elaphus*) from May to September, during the period of 2012 to 2019 in Tapada Nacional de Mafra (38°57′10″ N, 9°17′47.68″ W), district of Lisbon, west coast of Portugal. This nature reserve of 819 hectares is about 7 km from Mafra, Portugal. Mixed forests of eucalyptus (*Eucalyptus globulus*); cork oak (*Quercus suber*); maritime pine (*Pinus pinaster*); stone pine (*Pinus pinea*); and other less common tree species, bushes, and shrubs constitute the main vegetation. Within this habitat, fallow and red deer coexist with medium-sized mammals such as foxes and wild boar, as well as small mammals including shrews, mice, and several species of bats [60]. There are no livestock in the reserve which can be visited by the public on weekends.

The ticks in this study were collected from the deer as part of management of the reserve. Deer immobilization and tick collection were authorized by Tapada Nacional de Mafra-Cooperativa de Interesse Público de Responsabilidade Limitada (CIPRL), following the European Council Directive 97/62/EC of 27.10.1997 on the conservation of natural habitats and of wild fauna and flora, and conducted by qualified veterinarians from the non-profit nature conservation organization BeWild. The collected ticks were then kindly donated to the study by BeWild. Tissue and/or blood samples from these animals for pathogen analysis were not available. Ticks were received in polypropylene tubes containing 70% ethanol. They were then separated by sex and identified to species level following morphological and taxonomic keys [61]. All ticks were individually stored at 4 °C until molecular analysis.

### 5.2. Genomic DNA Extraction

Ticks were individually rinsed in sterile phosphate buffered saline (PBS) solution, before being disrupted and homogenized with pestles. Genomic DNA was extracted from each homogenized tick using the commercial kit Speedtools^®^ tissue DNA extraction (Biotools, Madrid, Spain) according to the manufacturer’s instructions with slight modifications. RNA was removed by RNase digestion (Roche Diagnostic GmbH, Germany). Quantification and purity of the DNA samples were determined by spectrophotometry with a NanoDrop ND-1000 spectrophotometer (Nucliber, Madrid, Spain). DNA samples were eluted in 100 μL of sterile water and stored at −20 °C for further analysis.

### 5.3. Detection of Piroplasms

Molecular methods for detecting the presence of piroplasms were based on amplification of part of the 18S rRNA gene. All ticks were tested using (i) the piroplasm real-time PCR and primers PIRO-A and PIRO-B [30,31] and (ii) a *B. microti* real-time PCR using the specific set of primers Bab2/Bab3 [32]. To unequivocally distinguish between *B. divergens* and *B. capreoli*, amplification of part of the COI gene was carried out following a previously described protocol [13]. A conventional PCR was optimized to amplify large fragments of the 18S rRNA gene using samples that were previously found positive for *B. divergens* and *Theileria* species. A nested PCR was also performed to amplify large fragments of the 18S rRNA gene using positive *B. microti* samples [35]. The set of primers were: 18SRNABABF1/18SRNABABR1 for *B. divergens*, BABGF2/18SRNABABR1 for *Theileria* spp., and Piro0F2/Piro6R2 and Piro1F2/Piro5R2 for *B. microti*. Table 3 shows all primers and expected sizes of the amplified products.

Each reaction was performed in a final volume of 50 μL containing 200–400 ng of DNA, 2X PrimeStar GXL Buffer (Takara Bio, Shiga, Japan), 800 μM of dNTPs mixture (Takara Bio), 1.25 U of PrimerStar GXL DNA polymerase (Takara Bio), 1 μL (20 mg/mL) of BSA DNAse Free (Roche, Basel Switzerland), and 0.3 μM of each primer.

DNA from *B. divergens* Bd Rouen 1987 and *B. microti* Gray (ATCC^®^ 30221™) were used as positive controls and water as negative control. To minimize contamination, false-positive samples, DNA extraction, PCR master-mix preparation, sample addition, and PCR reactions were performed in different biosafety cabinets in separate laboratories.

### 5.4. DNA Sequencing and Analysis

All positives qPCR and PCR products were separated on 1% and 2% agarose gels (Conda, Spain) stained with Pronasafe nucleic acid staining solution (10 mg/mL) (Conda, Spain) and visualized under UV illumination. The DNA bands were cut out of agarose gels under UV exposure, and purified using the mi-Gel Extraction Kit (Metabion international AG, Steinkirchen Germany). Both strands of DNA fragments were sequenced using an ABI PRISM 3730XL DNA Analyzer (Applied Biosystems, San Francisco, CA, USA). Primers and internal walking primers were used for sequencing *B. divergens*, *T. capreoli*, and *Theileria* sp. OT3 18S rRNA and cyt b genes. The subsequent electropherograms of the nucleotide sequences were manually inspected, corrected, and edited using ChromasPro program (McCarthy, Queensland, Australia) and LaserGene 12.1 program (DNAStar, Madison, WI, USA). All nucleotide sequences were compared to those deposited in the NCBI GenBank database using the BLAST algorithm (http://www.ncbi.nlm.nih.gov/BLAST, 27 November 2012). Nucleotide sequences longer than 200 bp were deposited in the NCBI GenBank under the following accession numbers: OL442188, OL442187, and OL442191.

### 5.5. Statistical Analysis

Statistical analyses were performed using the ABI-Prism 9 software. The level of significance was set at *p* < 0.05.

## Figures and Tables

**Table 1:** Species of ticks removed from fallow deer (*Dama dama*) and red deer (*Cervus elaphus*) in the Tapada Nature Reserve, Portugal.

Hosts	Ticks Species	Common Name	Relative Abundance in Portugal [29]	Zoonotic Pathogens * Transmitted	Tested (n)	Female	Male
Fallow deer	*Ixodes ricinus*	Castor bean tick	Common	*Babesia, Borrelia, Rickettsia, Anaplasma,* TBEV	377	291	86
	*Rhipicephalus sanguineus* s.l.	Brown dog tick	Common	*Rickettsia*	42	24	18
	*Hyalomma lusitanicum*	None	Locally common in south	None	22	15	7
	*Haemaphysalis punctata*	Red sheep tick	Sporadic distribution	None	15	13	2
	*Dermacentor marginatus*	Ornate sheep tick	Locally common	*Rickettsia*	6	5	1
	*Ixodes hexagonus*	Hedgehog tick	Common	None	2	2	-
Red deer	*I. ricinus*	As above	As above	As above	31	19	12
	*R. sanguineus* s.l.	As above	As above	As above	23	7	16
	*D. marginatus*	As above	As above	As above	2	1	1
Total					520	377	143

s.l.—sensu lato; TBEV—tick-borne encephalitis virus. * Most of these tick species have been implicated as carriers of the agent of Q-fever, *Coxiella burnetii,* but their role as vectors of this pathogen is unclear.

**Table 2:** *Babesia* spp. and *Theileria* spp. DNA detected in ticks collected from fallow deer and red deer.

Host	Tick species	Tested (n)	qPCR Result (n[%])									
			1 pathogen				2 pathogens					3 pathogens
												*Babesia*
												*microti*
												*+*
							*Babesia*	*Babesia*	*Babesia*	*Babesia*	*Theileria*	*Theileria*
							*Divergens*	*Divergens*	*microti*	*microti*	*capreoli*	*capreoli*
							*+*	*+*	*+*	*+*	*+*	*+*
			*Babesia divergens*	*Babesia microti*	*Theileria sp. OT3*	*Theileria capreoli*	*Babesia microti*	*Theileria sp. OT3*	*Theileria sp. OT3*	*Theileria capreoli*	*Theileria sp. OT3*	*Theileria sp. OT3*
Fallow deer	*I. ricinus*	377	5 (1.3)	14 (3.7)	22 (5.8)	6 (1.6)	2 (0.5)	1 (0.3)	6 (1.6)	2 (0.5)	1 (0.3)	1 (0.3)
	*R. sanguineus* s.l.	42	-	3 (7.1)	2 (4.8)	-	-	-	1 (2.4)	-	-	-
	*H. lusitanicum*	22	-	2 (9.1)	-	-	-	-	-	-	-	-
Red deer	*I. ricinus*	31	-	-	-	5 (16.1)	-	-	-	-	-	-
	*R. sanguineus* s.l.	23	-	-	1 (4.3)	1 (4.3)	-	-	-	-	-	-

**Table 3:** Targets, oligonucleotide primers, size of amplicons, and melting temperature of the primers used for the PCR detection of piroplasms in ticks removed from fallow deer and red deer.

Target Gene *	Primer Name	Nucleotide Sequence (5′-3′)	Product Size (bp)	Tm (°C)	Reference
18S	PIROA	AATACCCAATCCTGACACAGGG	408–430	62	[30,31]
rRNA	PIROB	TTAAATACGAATGCCCCCAAC			
	Bab2	GTTATAGTTTATTTGATGTTCGTTT	155–157	54	[32]
	Bab3	AAGCCATGCGATTCGCTAAT			
COI	Bdiv-F165	AGTGGAACTGGGTGGACATTGTAC	234	60	[13]
	Bdiv-R398	TACCGGCAATGACAAAAGTAG			
	BcapF165	AGTGGAACAGGATGGACGCTATAT	443	60	[13]
	Bcap-R607	GTCTGATTACCGAACACTTCC			
18S	18SRNABABF1	GCATGTCTAAGTACAAACTTTTTAC	1610	60	This study
rRNA	18SRNABABR1	AAGGTTCACAAGACTTCCCTAGGC			
	BABGF2	GTCTTGTAATTGGAATGATGG	1192	55	This study
	18SRNABABR1	AAGGTTCACAAGACTTCCCTAGGC			
	Piro0F2	GCCAGTAGTCATATGCTTGTCTTA	1702	60	[35]
	Piro6R2	CTCCTTCCTTTAAGTGATAAGGTTCAC			
	Piro1F2	CCATGCATGTCTTAGTATAAGCTTTTA	1670	60	[35]
	Piro5R2	CCTTTAAGTGATAAGGTTCACAAAACTT			
COI	Cox1F133	GGAGAGCTAGGTAGTAGTGGAGATAGG	1023	56	[35]
	Cox1R1130	GTGGAAGTGAGCTACCACATACGCTG			

18S rRNA- 18S ribosomal RNA, COI- cytochrome c oxidase subunit I, Tm- melting temperature.

## Data Availability

Not applicable.

## Supplementary Materials

The following are available online at https://www.mdpi.com/article/10.3390/pathogens11020222/s1, Figure S1: *Babesia microti* detection by PCR and DNA sequencing. Table S1: Tick species removed from fallow deer, showing distribution of piroplasm-infected ticks. Table S2: Tick species removed from red deer, showing distribution of piroplasm-infected ticks.
