# Supplementary material for: Babesia and Theileria Identification in Adult Ixodid Ticks from Tapada Nature Reserve, Portugal
Source: Pathogens. 2022 Feb 8;11(2):222. doi: 10.3390/pathogens11020222 (PMC8876925; doi:10.3390/pathogens11020222)
Supplement: Supplementary file 1 [file pathogens-11-00222-s001.zip › New_Supplementary_Table S1.pdf]

Suppl. Table S1. Tick species removed from fallow deer, showing distribution of piroplasm-infected ticks. Infected ticks were PCR positive for one, two or three pathogens.

**Fallow deer**	**tick**	**n**	***Babesia divergens***	***Babesia microti***	***Theileria* sp. OT3**	***Theileria capreoli***	***Babesia divergens* + *Babesia microti***	***Babesia divergens* + *Theileria* sp. OT3**	***Babesia microti* + *Theileria* sp. OT3**	***Babesia microti* + *Theileria capreoli***	***Theileria capreoli* + *Theileria* sp. OT3**	***Babesia microti* + *Theileria capreoli* + *Theileria* sp. OT3**
1	*I. ricinus*	4	-	-	-	-	-	-	-	-	-	-
2	*R. sanguineus* s.l.	5	-	-	-	-	-	-	1	-	-	-
	H. lusitanicum	1	-	-	-	-	-	-	-	-	-	-
3	*I. ricinus*	1	-	-	-	-	-	-	-	-	-	-
	H. lusitanicum	1	-	1	-	-	-	-	-	-	-	-
6	*H. lusitanicum*	2	-	1	-	-	-	-	-	-	-	-
7	*I. ricinus*	3	-	-	1	-	-	-	-	-	-	-
	H. punctata	4	-	-	-	-	-	-	-	-	-	-
	D. marginatus	1	-	-	-	-	-	-	-	-	-	-
8	*I. ricinus*	6	-	-	-	-	-	-	-	-	-	-
9	*I. ricinus*	6	-	1	-	-	-	-	1	-	-	-
10	*I. ricinus*	8	-	-	-	-	-	-	-	-	-	-
	H. lusitanicum	2	-	-	-	-	-	-	-	-	-	-
	H. punctata	3	-	-	-	-	-	-	-	-	-	-
11	*I. ricinus*	3	-	-	-	-	-	-	-	-	-	-
12	*I. ricinus*	3	-	-	-	-	-	-	-	-	-	-
	H. lusitanicum	6	-	-	-	-	-	-	-	-	-	-
13	*I. ricinus*	20	1	1	-	1	-	-	-	-	-	-
14	*I. ricinus*	2	-	-	1	-	-	-	-	-	-	-
	R. sanguineus s.l.	1	-	-	-	-	-	-	-	-	-	-
	H. lusitanicum	1	-	-	-	-	-	-	-	-	-	-
15	*I. ricinus*	14	-	-	-	-	-	-	-	-	-	-

16	*I. ricinus*	10	1	-	-	-	-	-	-	-	-	-
	R. sanguineus s.l.	1	-	-	-	-	-	-	-	-	-	-
	I. hexagonus	1	-	-	-	-	-	-	-	-	-	-
17	*I. ricinus*	10	-	-	-	-	-	-	-	-	-	-
18	*I. ricinus*	14	-	-	2	-	-	-	-	-	-	-
19	*I. ricinus*	2	-	-	-	-	-	-	-	-	-	-
20	*I. ricinus*	1	-	-	-	1	-	-	-	-	-	-
	R. sanguineus s.l.	1	-	-	-	-	-	-	-	-	-	-
21	*I. ricinus*	10	-	-	-	-	-	-	-	-	-	-
22	*I. ricinus*	5	-	-	1	-	-	-	-	-	-	-
23	*I. ricinus*	19	-	1		-	-	-	-	-	-	-
24	*I. ricinus*	5	-		3	-	-	-	-	-	-	-
	R. sanguineus s.l.	1	-	-	-	-	-	-	-	-	-	-
25	*I. ricinus*	3	-	-	-	-	-	-	-	-	-	-
	H. punctata	1	-	-	-	-	-	-	-	-	-	-
	I. hexagonus	1	-	-	-	-	-	-	-	-	-	-
26	*I. ricinus*	5	-	-	-	-	-	-	-	-	-	-
	H. punctata	1	-	-	-	-	-	-	-	-	-	-
27	*I. ricinus*	1	-	-	-	-	-	-	-	-	-	-
	R. sanguineus s.l.	1	-	-	-	-	-	-	-	-	-	-
28	*I. ricinus*	10	-	-	-	1	-	-	-	-	-	-
	D. marginatus	1	-	-	-	-	-	-	-	-	-	-
29	*I. ricinus*	11	-	-	1	-	-	-	-	-	-	-
	H. punctata	1	-	-	-	-	-	-	-	-	-	-
30	*I. ricinus*	11	-	-	1	-	-	-	-	-	-	-
31	*I. ricinus*	7	-	-	2	-	-	-	-	-	-	-
32	*I. ricinus*	7	-	-	1	-	-	-	-	-	-	-
33	*R. sanguineus* s.l.	4	-	-	-	-	-	-	-	-	-	-
	H. lusitanicum	1	-	-	-	-	-	-	-	-	-	-
34	*I. ricinus*	3	-	-	-	-	-	-	-	-	-	-
35	*I. ricinus*	7	-	1	-	-	-	-	-	-	-	-
36	*I. ricinus*	7	-	-	-	-	-	-	-	-	-	-
	H. punctata	1	-	-	-	-	-	-	-	-	-	-

37	*I. ricinus*	8	-	-	-	-	-	-	-	-	-	-
38	*I. ricinus*	11	-	-	-	-	-	-	-	-	-	-
39	*I. ricinus*	4	-	-	-	-	1	-	-	-	-	-
40	*I. ricinus*	13	1	2	-	-	-	-	-	-	1	-
41	*I. ricinus*	5	-	-	-	-	-	-	-	-	-	-
	R. sanguineus s.l.	1	-	-	-	-	-	-	-	-	-	-
	H. lusitanicum	1	-	-	-	-	-	-	-	-	-	-
42	*I. ricinus*	1	-	-	-	-	-	-	-	-	-	-
	R. sanguineus s.l.	6	-	1	1	-	-	-	-	-	-	-
43	*I. ricinus*	1	-	-	-	-	-	-	-	-	-	1
	R. sanguineus s.l.	1	-	-	-	-	-	-	-	-	-	-
	H. lusitanicum	3	-	-	-	-	-	-	-	-	-	-
44	*R. sanguineus* s.l.	3	-	1	-	-	-	-	-	-	-	-
45	*I. ricinus*	5	-	1	2	-	-	-	1	-	-	-
	R. sanguineus s.l.	1	-	-	1	-	-	-	-	-	-	-
46	*I. ricinus*	11	-	2	-	-	-	-	1	1	-	-
47	*I. ricinus*	8	-	2	-	1	-	-	-	-	-	-
	R. sanguineus s.l.	3	-	1	-	-	-	-	-	-	-	-
	H. punctata	1	-	-	-	-	-	-	-	-	-	-
48	*I. ricinus*	14	-	-	2	-	-	-	-	-	-	-
	R. sanguineus s.l.	1	-	-	-	-	-	-	-	-	-	-
49	*I. ricinus*	1	-	-	1	-	-	-	-	-	-	-
	H. punctata	2	-	-	-	-	-	-	-	-	-	-
50	*I. ricinus*	6	-	-	1	-	-	1	-	-	-	-
	R. sanguineus s.l.	6	-	-	-	-	-	-	-	-	-	-
51	*I. ricinus*	5	-	-	1	-	-	-	-	-	-	-
	R. sanguineus s.l.	4	-	-	-	-	-	-	-	-	-	-
52	*I. ricinus*	8	1	-	2	-	-	-	1	-	-	-
53	*I. ricinus*	7	1	-	-	-	-	-	-	-	-	-
54	*I. ricinus*	11	-	1	-	-	1	-	1	-	-	-
55	*I. ricinus*	9	-	-	-	-	-	-	-	-	-	-
56	*I. ricinus*	8	-	1	-	-	-	-	-	-	-	-
57	*I. ricinus*	5	-	-	-	-	-	-	-	1	-	-

58	*H. lusitanicum*	3	-	-	-	-	-	-	-	-	-	-
59	*I. ricinus*	7	-	-	-	-	-	-	-	-	-	-
	H. lusitanicum	1	-	-	-	-	-	-	-	-	-	-
	D. marginatus	2	-	-	-	-	-	-	-	-	-	-
60	*I. ricinus*	1	-	-	-	-	-	-	-	-	-	-
	D. marginatus	1	-	-	-	-	-	-	-	-	-	-
61	*I. ricinus*	3	-	1	-	-	-	-	1	-	-	-
	R. sanguineus s.l.	1	-	-	-	-	-	-	-	-	-	-
62	*R. sanguineus* s.l.	1	-	-	-	-	-	-	-	-	-	-
63	*I. ricinus*	1	-	-	-	-	-	-	-	-	-	-
	H. punctata	1	-	-	-	-	-	-	-	-	-	-
65	*I. ricinus*	1	-	-	-	-	-	-	-	-	-	-
66	*I. ricinus*	1	-	-	-	-	-	-	-	-	-	-
67	*D. marginatus*	1	-	-	-	-	-	-	-	-	-	-
68	*I. ricinus*	1	-	-	-	-	-	-	-	-	-	-
69	*I. ricinus*	1	-	-	-	-	-	-	-	-	-	-
70	*I. ricinus*	1	-	-	-	1	-	-	-	-	-	-
71	*I. ricinus*	1	-	-	-	1	-	-	-	-	-	-
