# Supplementary material for: Babesia and Theileria Identification in Adult Ixodid Ticks from Tapada Nature Reserve, Portugal
Source: Pathogens. 2022 Feb 8;11(2):222. doi: 10.3390/pathogens11020222 (PMC8876925; doi:10.3390/pathogens11020222)
Supplement: Supplementary file 1 [file pathogens-11-00222-s001.zip › New_Supplementary_Table S2.pdf]

Suppl. Table S2. Tick species removed from red deer, showing distribution of piroplasm-infected ticks. Infected ticks were PCR positive for either *Theileria* sp. OT3 or *T. capreoli*.

Red deer	tick	n	*Theileria* sp. OT3	*Theileria capreoli*
1	*I. ricinus*	10	-	1
2	*I. ricinus*	2	-	-
	R. sanguineus s.l.	1	-	-
3	*I. ricinus*	5	-	-
	R. sanguineus s.l.	2	-	-
4	*I. ricinus*	1	-	1
	R. sanguineus s.l.	4	-	-
	D. marginatus	1	-	-
5	*I. ricinus*	6	-	3
6	*I. ricinus*	1	-	-
	R. sanguineus s.l.	6	-	-
7	*I. ricinus*	2	-	-
	R. sanguineus s.l.	1	1	-
	D. marginatus	1	-	-
8	*R. sanguineus* s.l.	6	-	-
9	*I. ricinus*	2	-	-
10	*R. sanguineus* s.l.	3	-	1
11	*I. ricinus*	1	-	-
12	*I. ricinus*	1	-	-
