# Supplementary material for: Babesia and Theileria Identification in Adult Ixodid Ticks from Tapada Nature Reserve, Portugal
Source: Pathogens. 2022 Feb 8;11(2):222. doi: 10.3390/pathogens11020222 (PMC8876925; doi:10.3390/pathogens11020222)
Supplement: Supplementary file 1 [file pathogens-11-00222-s001.zip › Supplementary_Figure S1.pdf]

```
B. microti 157 bp GTTATAGTTTATTTGATGTTCGTTTTACATGGATAACCGTGGTAATTCTAGGGCTAATAC

B. microti 155 bp GTTATAGTTTATTTGATGTTCGTTTTACATGGATAACCGTGGTAATTCTAGGGCTAATAC

                  ************************************************************


B. microti 157 bp ATGCTCGAGGCGCGTTTTACGCGTGGCGTTTATTAGACTTTAACCAGCCCCTTTGGGTAA

B. microti 155 bp ATGCTCGAGGCGCGTTTT-CGCGTGGCGTTTATTAGACTTTAACCAA-CCCTTCGGGTAA

                  ****************** ***************************  ***** ******


B. microti 157 bp TCGGTGATTCATAATAAATTAGCGAATCGCATGGCTT

B. microti 155 bp TCGGTGATTCATAATAAATTAGCGAATCGCATGGCTT

                  *************************************
```

Figure S1: *Babesia microti* detection by PCR and DNA sequencing.
